# #GERIATRICS: A 7-YEAR TWITTER ANALYSIS

**DOI:** 10.1093/geroni/igz038.626

**Published:** 2019-11-08

**Authors:** Brenna M Parker, Megan Walker, Jeanette Ross

**Affiliations:** 1 UTHSA Long School of Medicine, San Antonio, Texas, United States; 2 UT Health Science Center San Antonio, San Antonio, Texas, United States; 3 South Texas Veterans Health Care System, San Antonio, Texas, United States

## Abstract

The use of social media platforms as an educational tool to promote awareness has become increasingly popular as technology advances. Twitter is a microblogging, social media platform in which users share short, text-based posts (“tweets”) that can contain hyperlinked articles, web-pages, pictures, and more. 79% of the 336 million current monthly twitter users are international, suggesting Twitter serves as a tool allowing international connection via the rapid spread of information worldwide. Simplur Signals (Simplur LLC) was used to perform a retrospective analysis of the use of #Geriatrics on Twitter. Data was collected from Oct. 13th, 2010 through Jun. 5th, 2018. Spam and unknown accounts were excluded from the data set before analysis. Manual analysis was performed to qualitatively assess tweet content of the top 200 Retweets by Impressions. A total of 65,002 tweets were shared during the selected time frame. Tweet activity rose to a high in Year 5 (17,206) but has declined since. The majority of the top 100 influencers were doctors (57.4%). Regarding tweet content, most discussions focus on increasing awareness and promoting advocacy (30%) as well as sharing research related to the practice of geriatrics (23.5%). With its widespread use and lack of international boundaries, Twitter serves as an effective platform in informing and increasing awareness about geriatrics and other medical specialties.

